# Model to predict cause‐specific mortality in patients with olfactory neuroblastoma: a competing risk analysis

**DOI:** 10.1186/s13014-021-01784-8

**Published:** 2021-06-10

**Authors:** Lipin Liu, Qiuzi Zhong, Ting Zhao, Dazhi Chen, Yonggang Xu, Gaofeng Li

**Affiliations:** Department of Radiation Oncology, Beijing Hospital, National Center of Gerontology, Institute of Geriatric Medicine, Chinese Academy of Medical Sciences, Beijing, China

**Keywords:** Olfactory neuroblastoma, Competing risk analysis, Cause‐specific mortality, Nomogram

## Abstract

**Purpose:**

The main objective of this study was to evaluate the cumulative incidence of cause-specific mortality and other causes of mortality for patients with olfactory neuroblastoma (ONB). The secondary aim was to model the probability of cause-specific death and build a competing risk nomogram to predict cause-specific mortality for this disease.

**Methods:**

Patients with ONB from 1975 to 2016 were identified from the Surveillance, Epidemiology, and End Results database. We estimated the cumulative incidence function (CIF) for cause-specific mortality and other causes of mortality, and constructed the Fine and Gray’s proportional subdistribution hazard model, as well as a competing-risk nomogram based on Fine and Gray’s model, to predict the probability of cause-specific mortality for patients with ONB.

**Results:**

After data selection, 826 cases were included for analysis. Five-year cumulative incidence of cause-specific mortality was 19.5% and cumulative incidence of other causes of mortality was 11.3%. Predictors of cause-specific mortality for ONB included tumor stage, surgery and chemotherapy. Age was most strongly predictive of other causes of mortality: patients aged > 60 years exhibited subdistribution hazard ratios of 1.063 (95 % confidence interval [CI] 1.05–1.08; *p* = 0.001). The competing risk nomogram for cause-specific mortality was well-calibrated, and had good discriminative ability (concordance index = 0.79).

**Conclusions:**

We calculated the CIF of cause-specific mortality and other causes of mortality in patients with the rare malignancy ONB. We also built the first competing risk nomogram to provide useful individualized predictive information for patients with ONB.

## Introduction

Olfactory neuroblastoma (ONB) is an uncommon malignancy that arises from the olfactory epithelium and represents approximately 3% of all sinonasal malignancies [1, 2]. With improvement in pathological recognition, the incidence of ONB is steadily increasing in recent years [3]. Regarding age distribution, recent studies would suggest the peak in the fifth to sixth decades demonstrating a unimodal age distribution [4, 5]. Based on several retrospective reviews [6–10] and meta-analyses[2, 11], surgical resection combined with radiotherapy is the current best treatment modality resulting in prolonged 5 year OS ranging from 65 to 75% [1, 2, 12–14]. Given the high incidence in the elderly as well as the relatively long-term survival for patients with ONB, a considerable number of patients may die of other non-cancer causes, especially for the elderly. As a result, presentation of other causes of mortality should be taken into account when evaluating the prognosis for ONB.

Although many studies have reported the prognosis of ONB, most of them focused on the overall survival and cancer-specific survival based on the Kaplan-Meier method and COX proportional approach. However, these conventional statistical methods may be inappropriate for the fact that they consider competing events as independent censoring and may overestimate the incidence of cause-specific mortality. In contrast, competing risk method based on the subdistribution hazard model could better discriminate the effects of risk factors on specific events. As a result, competing event model is recommended in the presence of competing causes of mortality [15]. Competing risk nomogram for breast cancer, thyroid cancer and prostate cancer [16–18] have been developed in recent years. However, no comprehensive nomogram for ONB based on a competing risk model has been described.

In this study, we reviewed all ONB cases registered in the Surveillance, Epidemiology, and End Results (SEER) database from 1975 to 2016 and conducted a competing risk analysis. We calculated the cumulative incidence function (CIF) for cause-specific mortality and other causes of mortality, and developed a model to predict cause-specific mortality. We also established a competing risk nomogram for clinicians to predict probability of cause-specific mortality for ONB.

## Methods

The study population was obtained from the records of the SEER 18 program released in 2018 of the National Cancer Institute. Patients diagnosed with ONB as a first primary malignancy between 1975 and 2016 were selected for the study. Since SEER data were anonymized and openly accessible, institutional review board approval was not needed for our study.

Tumor site and histology were grouped according to the International Classification of Diseases for Oncology, third edition (ICD-O-3). The study cohort consisted of patients with ICD-O-3 histology codes 9522/2 (ONB) and ICD-O-3 site codes as follows: C30.0 (nasal cavity), C31.0 (maxillary sinus), C31.1 (ethmoid sinus), C31.2 (frontal sinus), C31.3 (sphenoid sinus), C31.8 (overlapping lesion of accessory sinus), and C31.9 (accessory sinus, not otherwise specified [NOS]). Patients with histologically confirmed ONB were included and patients diagnosed at autopsy or by death certificate were excluded. Patients with complete data on race, age at diagnosis, gender, primary site, SEER stage, treatment (including surgery, radiotherapy, and chemotherapy) and SEER cause of death (COD) record were included in this study. Variables in the analysis included race, age, sex, primary site, SEER stage, surgery, radiotherapy and chemotherapy. Staging was grouped into three broad categories according to the SEER historical staging classification: localized, regional, and distant disease. Age was grouped as ≤ 60 years and > 60 years.

Cause-specific mortality and other causes of mortality were considered as two competing events. We used the cumulative incidence function (CIF) to describe the probability of mortality. Gray’s test was conducted to test the CIF difference between groups [19]. We performed Fine and Gray proportional subdistribution hazards regression to build the competing risk model [20]. We built a competing risk nomogram based on Fine and Gray’s model to predict cause-specific mortality for patients with ONB [21]. Concordance index (c-index) value was used to assess discrimination and calibration plot was used to evaluate calibration via a bootstrap approach with 1000 resamples. All analyses were performed using R version 3.6.3 software (). The R packages cmprsk, rms, and mstate were used for building the model and nomogram and the package pec was used for evaluating model performance. All reported *p* values were two-sided, and the level of significance was set at 0.05.

## Results

A total of 826 patients with histologically confirmed ONB were identified in this analysis. Patients’ characteristics are shown in Table 1. The median age at diagnosis was 54 years. The majority of patients were ≤ 60 years old (65.1%), male (57.1%) and white (80.9%). The most common primary site was nasal cavity (77.6%). As to tumor stage distribution, 24.1%, 43.2% and 32.7% of patients presented with localized, regional and distant disease, respectively. Most patients (95.4%) were treated with surgery. A total of 72% of patients underwent radiotherapy, and only 30.8% of patients received chemotherapy.

**Table 1:** Five-year cumulative incidences of mortality among patients with ONB

Characteristic	N (%)	Event (%)	Cause-specific mortality		Other causes of mortality	
			5-year (%)	*p* value	5-year (%)	*p* value
Age (years)				0.47		< 0.001
≤ 60	538 (65.1)	194 (51.5)	17.8		5.0	
> 60	288 (34.9)	183 (48.5)	22.7		23.1	
Sex				0.02		0.10
Male	472 (57.1)	233 (61.8)	21.1		13.7	
Female	354 (42.9)	144 (38.2)	17.4		8.1	
Race				0.41		0.98
White	668 (80.9)	300 (79.6)	18.8		11.3	
Non-white	158 (19.1)	77 (20.4)	22.6		11.2	
Primary site				0.84		0.47
Nasal cavity	641(77.6)	288 (76.4)	18.5		11.2	
Nasal sinus	185(22.4)	89 (23.6)	22.7		11.4	
SEER stage				< 0.001		0.52
Localized	199 (24.1)	156 (41.4)	3.7		6.9	
Regional	357 (43.2)	162 (43.0)	17.2		11.1	
Distant	270 (32.7)	59 (15.6)	34.7		15.0	
Surgery				< 0.001		< 0.001
Yes	705 (85.4)	286 (75.9)	15.8		8.7	
No	121 (14.6)	91 (24.1)	40.4		26.0	
Radiotherapy				0.20		0.04
Yes	595 (72.0)	272 (72.1)	20.0		10.6	
No	231 (28.0)	105 (27.9)	17.9		13.0	
Chemotherapy				< 0.001		0.19
Yes	254 (30.8)	144 (38.2)	36.6		9.5	
No	572 (69.2)	233 (61.8)	12.0		12.1	

*ONB* olfactory neuroblastoma

**Fig. 1:**
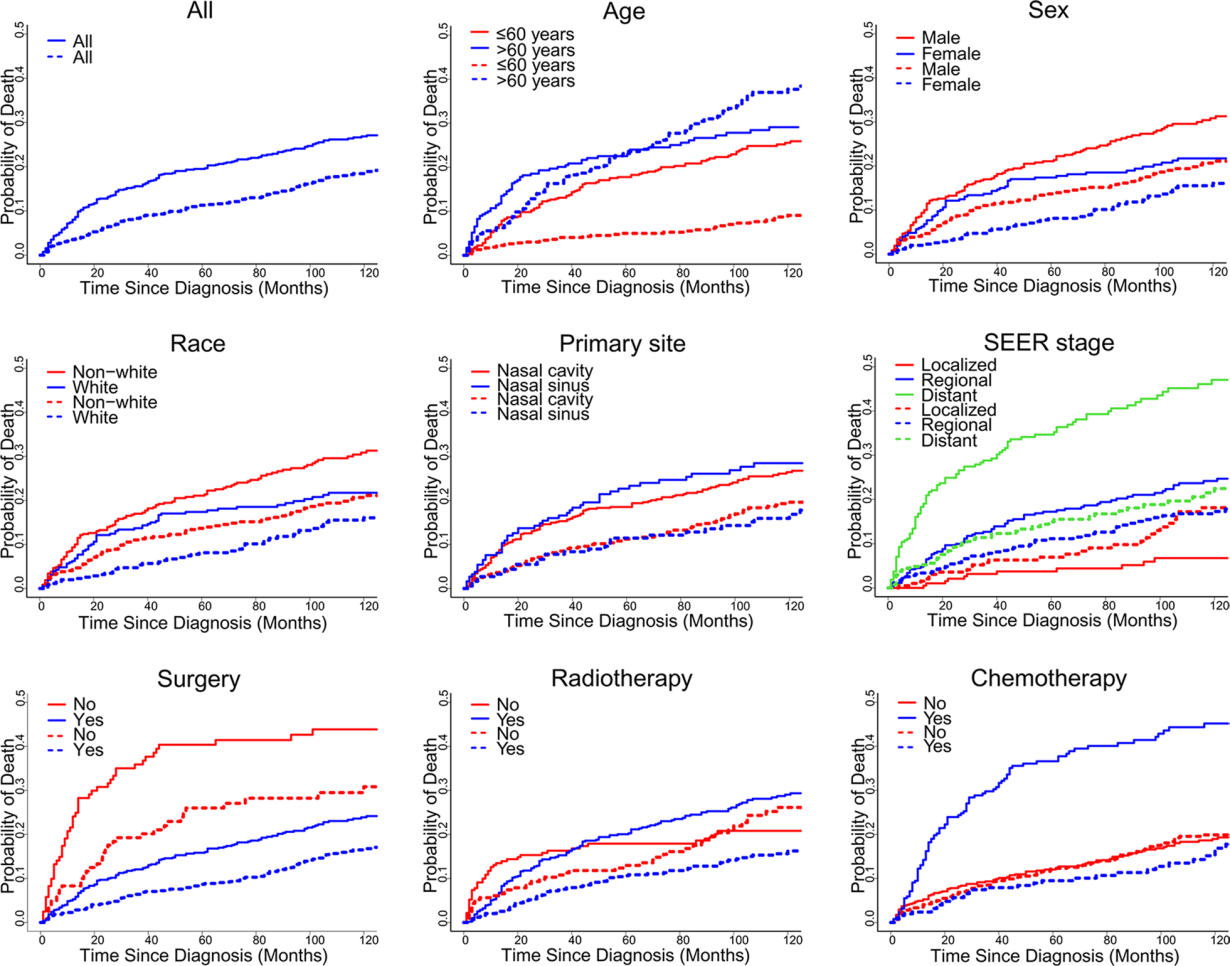
Cumulative incidence curves of deaths according to patient characteristics (solid line represents cause-specific death; dotted line represents other cause of death)

The median follow-up was 122 months (interquartile range 61–188 months). At last contact, 449 patients (54.3%) had been censored, and 377 patients (45.6%) had died, including 223 deaths (27.0%) from ONB and 154 (18.6%) from other causes. The 5-year estimates of the cumulative incidence of cause-specific mortality and other causes of mortality according to patient characteristics are listed in Table 1. The corresponding CIF curves are shown in Fig. 1. Five-year cumulative incidence of cause-specific mortality was 19.5% and cumulative incidence of other causes of mortality was 11.3%. The competing risk analysis showed increased cumulative incidence of cause-specific mortality for patients with male (*p* = 0.02), advanced stage (*p* < 0.001), no surgery (*p* < 0.001) and chemotherapy treatment (*p* < 0.001). Age > 60 years (*p* < 0.001), no surgery (*p* < 0.001) and no radiotherapy (*p* = 0.04) were significantly associated with increased cumulative incidence of other causes of mortality.

The proportional subdistribution hazard models of probabilities of cause-specific mortality for patients with ONB are presented in Table 2. Tumor stage, surgery and chemotherapy were independent predictors of cause-specific mortality. Compared with localized disease, patients with regional disease (SHR = 2.67, 95% CI 1.64–4.34, *p* < 0.001) and distant disease (SHR = 5.28, 95% CI 3.11–8.95, *p* < 0.001) experienced higher risk of cause-specific mortality. Increased probability of mortality from ONB was observed in patients who did not receive surgery (SHR = 2.67, 95% CI 1.64–4.34, *p* < 0.001). Patients without chemotherapy were at lower risk of cause-specific mortality compared with those who underwent chemotherapy (SHR = 0.52, 95% CI 0.38–0.71, *p* < 0.001). Age was significantly predictive of other causes of mortality; compared with patients < 60 years, a significantly higher cumulative incidence of other causes of mortality was observed in patients ≥ 60 years (SHR = 1.06, 95% CI 1.05–1.08, *p* = 0.001).

**Table 2:** Proportional subdistribution hazard models of probabilities of mortality for patients with ONB

Characteristic	Cause-specific mortality			Other causes of mortality		
	SHR	95% CI	*p* value	SHR	95% CI	*p* value
Age (years)						
≤ 60	Ref		0.75	Ref		
> 60	1.05	0.78–1.43		1.06	1.05–1.08	0.001
Sex			0.21			0.07
Male	Ref			Ref		
Female	0.83	0.63–1.11		0.75	0.55–1.02	
Race			0.75			0.63
White	Ref			Ref		
Non-white	1.06	0.76–1.48		1.10	0.74–1.64	
Primary site			0.19			0.10
Nasal cavity	Ref			Ref		
Nasal sinus	1.28	0.89–1.84		0.71	0.47–1.07	
SEER stage						
Localized	Ref			Ref		
Regional	2.67	1.64–4.34	< 0.001	0.85	0.58–1.25	0.42
Distant	5.28	3.11–8.95	< 0.001	0.80	0.52–1.25	0.33
Surgery			0.02			0.34
Yes	Ref					
No	1.53	1.07–2.19		1.24	0.80–1.92	
Radiotherapy			0.22			0.15
Yes	Ref					
No	1.25	0.88–1.77		1.32	0.91–1.91	
Chemotherapy			< 0.001			0.86
Yes	Ref					
No	0.52	0.38–0.71		0.96	0.62–1.48	

*ONB* olfactory neuroblastoma, *SHR* subdistribution hazard ratio, *CI* confidence interval

**Fig. 2:**
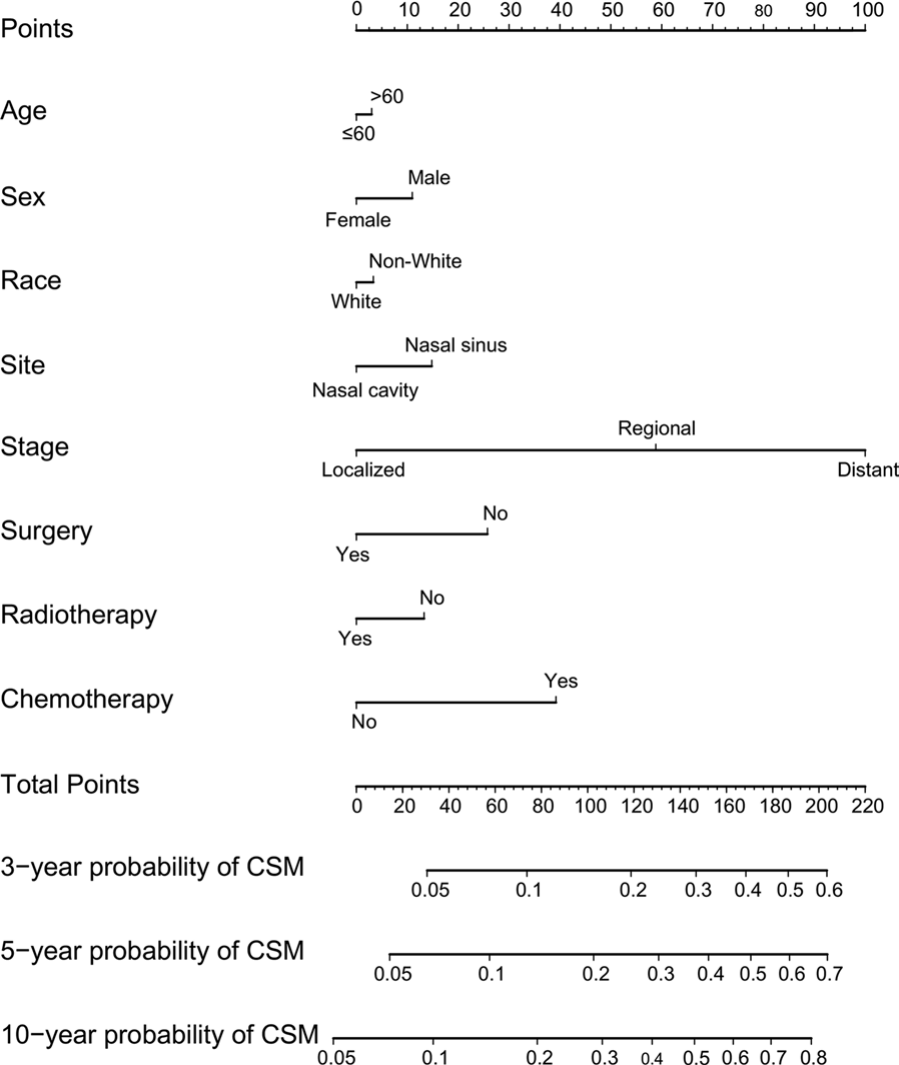
Nomogram for predicting 3-, 5- and 10-year probabilities of CSM in patients with ONB. *ONB* olfactory neuroblastoma, *CSM* cause-specific mortality

**Fig. 3:**
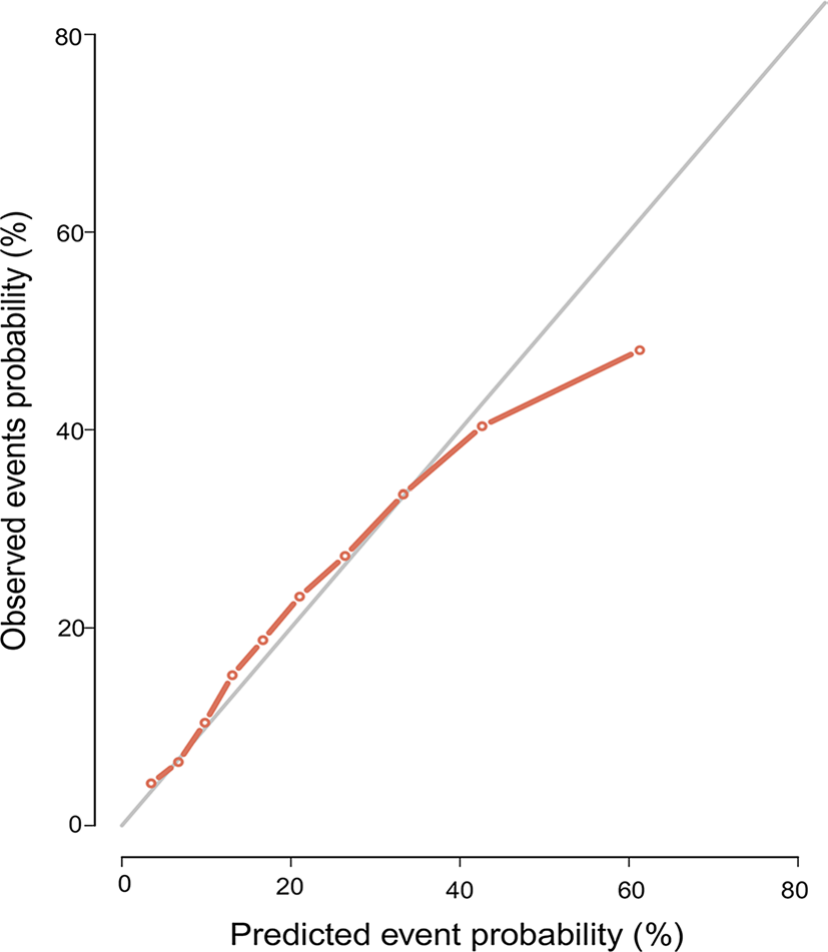
Calibration plot for cause specific mortality nomogram of ONB. The solid line represents equality between the predicted and observed probabilities. *ONB* olfactory neuroblastoma

The nomogram shown in Fig. 2 was constructed based on Fine and Gray’s model. This nomogram can predict probability of 3-, 5- and 10-year cause-specific mortality for patients with ONB by calculating the sum of points according to each patient’s characteristics. The model showed relatively good discriminative ability, with c-index of 0.79. The calibration plot of the CIF is shown in Fig. 3. The points close to the 45-degree line indicate good agreement between the nomogram-predicted probabilities and actual observations.

## Discussion

This study evaluated the mortality of patients with ONB registered in the SEER database from 1975 to 2016. Five-year cumulative incidence of cause-specific mortality and other causes of mortality were 19.5% and 11.3%, respectively. To our knowledge, this study is the first to develop competing risk nomogram based on the proportional subdistribution hazard approach to predict ONB specific mortality.

Previous studies have investigated the prognostic factors for ONB with inconsistent results. The widely recognized prognostic factors are tumor stage and Hyams’ histopathological grading [12, 13, 22], which is consistent with our results that advanced stage predict unfavorable cause-specific survival. However, Hyams’ grading data was not available in SEER database. The prognostic role of age for ONB is contradictory. Studies by Kadish [23] and Bisognoetal [24] demonstrated that ONB behaves differently among different age groups and tends to have a more aggressive presentation in the younger groups than in adults. Nevertheless, Eich et al. [25] suggested that children and adolescents with ONB could benefit from multimodal treatment with 5 year OS at 73%, which is comparable to adult population. Recently, a population based study by Yin et al. [5] analyzed patients with ONB in SEER database registered from 1973 to 2014 using the Cox method. The study concluded that the risk of overall mortality and cause-specific mortality increased with age by 3.1% and 1.6% per year. However, the study based on Cox method may overestimate the incidence of cause-specific mortality. In our competing risk analysis, advancing age was a strong predictor of other causes of mortality but was not associated with cause-specific mortality. Our results underscores the significance of treating other causes of mortality as a competing event in elderly patients for the fact that older patients were at greater risk of severe comorbidities and other causes of mortality.

Given the rarity of ONB, no prospective randomized clinical studies have been conducted to establish the agreed standard-of-care treatment algorithm. To date, multimodality treatment combining surgery and radiotherapy is the most widely accepted treatment approach [2, 4, 26]. In our study, surgery was associated with improved cause-specific survival, which was in consistent to previous studies indicating surgery is the mainstay treatment for ONB [26, 27]. Radiotherapy has been demonstrated to play an important role in the management of ONB. For Kadish stage A/B disease, some retrospective studies suggested that definitive radiotherapy alone could provide comparable treatment outcome with combination of surgery and radiation [22, 28, 29]. Post-operative and pre-operative radiotherapy could reduce local recurrence and improve survival by reducing local recurrence or increasing complete resection rate, especially for patients with advanced disease (Kadish stage C/D) [26, 30]. However, there is no consensus on the timing of radiotherapy when combined with surgery. The role of chemotherapy for ONB is contradictory. In our study, chemotherapy was associated with inferior cause-specific survival. This result regarding the role of chemotherapy should be interpreted with caution for the reason that lesions treated with chemotherapy had more advanced stage or higher risk of local recurrence and distant metastasis.

In our study, the five-year cumulative incidence of other causes of mortality was over half of cause-specific mortality (11.3% and 19.5%). As a result, competing causes of mortality represent a critical consideration when evaluating prognosis for decision making and patient counseling. To date, competing risk nomograms have been developed for common cancers such as breast cancer, thyroid cancer and prostate cancer [16–18]. To the best our knowledge, this is the first study to present CIF of cause-specific mortality and competing risk of mortality for ONB. Furthermore, this is the first attempt to establish a competing risk nomogram to predict cause-specific mortality for ONB. The strengths of our study include the population-based design, the large sample size and simplicity of competing risk nomogram model. Given the rarity of ONB, the population based SEER database can provide sufficient sample size to evaluate prognosis and develop an accurate predictive model. The nomogram can predict individual survival probability for specific outcomes at certain time points. Our nomogram by integrating a few prognostic factors showed good predictive ability, which would help doctors to make accurate individual prognosis estimates.

This study has several limitations that must be taken into account. First, some important prognostic factors such as Hyams’ grade, intracranial extension and surgical margin were unavailable in the SEER dataset. Inclusion of these prognostic factors will be a major part of our future research. In addition, patient data were collected from cases in SEER database diagnosed over the course of approximately 40 years. Pathological interpretation as well as treatment modalities including surgical technique, radiation technique and chemotherapy regimen have experienced massive changes in the past four decades. This model should be re-validated by patient data in recent years. Furthermore, we used internal validation by bootstrap approach to evaluate nomogram model performance. Although the nomogram showed good performance in cause-specific mortality prediction, external validation in other populations is still needed to estimate model accuracy.

## Conclusions

In the present study, we calculated the CIF of cause-specific mortality and other causes of mortality for patients with ONB using a large, population-based cohort from the SEER database. We also modeled the probability of cause-specific mortality for ONB by the proportional subdistribution hazard approach, as well as built a competing risk nomogram to calculate the 3-, 5- and 10-year cause specific mortality. Our nomogram showed a relatively good performance and could be a practical tool to predict individual prognosis. However, further external validation is still needed.

## Data Availability

The datasets used during the present study are available from the corresponding author upon reasonable request.

## Abbreviations

CIF: Cumulative incidence function
c-index: Concordance index
COD: Cause of death
NOS: Not otherwise specified
SHR: Subdistribution hazard ratio
SEER: Surveillance, Epidemiology, and End Results
ONB: Olfactory neuroblastoma
